# Safety and Long-Term Efficacy of Drug-Coated Balloon Angioplasty following Rotational Atherectomy for Severely Calcified Coronary Lesions Compared with New Generation Drug-Eluting Stents

**DOI:** 10.1155/2019/9094178

**Published:** 2019-03-13

**Authors:** Katsumi Ueno, Norihiko Morita, Yoshinobu Kojima, Hiroshi Takahashi, Masanori Kawasaki, Ryuta Ito, Hiroki Kondo, Shingo Minatoguchi, Tamami Yoshida, Yasumasa Hashimoto, Tomohiko Tatsumi, Tomoya Kitamura

**Affiliations:** ^1^Department of Cardiology, Matsunami General Hospital, Gifu, Japan; ^2^Department of Nephrology, Fujita Health University School of Medicine, Aichi, Japan; ^3^Department of Cardiology, Gifu University Graduate School of Medicine, Gifu, Japan; ^4^Department of Cardiology, Nagoya Kyoritsu Hospital, Aichi, Japan

## Abstract

**Objectives:**

This study sought to assess the safety and long-term efficacy of drug-coated balloons (DCB) following aggressive intracoronary image-guided rotational atherectomy (iRA) for severe coronary artery calcification (CAC), and to compare this strategy with new generation drug-eluting stents (nDES) following iRA.

**Background:**

Ischemic events following the treatment of CAC is still relatively high. Thus, more innovative strategies are required.

**Methods:**

We evaluated 123 consecutive patients (166 lesions) with de novo CAC undergoing an iRA (burr size; 0.7 of the mean reference diameter by intracoronary imaging) followed by DCB (DCB-iRA; 54 patients, 68 lesions) or nDES (nDES-iRA; 69 patients, 98 lesions). Follow-up angiography was obtained at > 6 months.

**Results:**

The target vessels (right coronary and circumflex), bifurcation (67.6% versus 47.9%), reference diameter (2.28mm versus 2.49mm), and lesion length (11.89mm versus 18.78mm) were significantly different between the two groups. The median follow-up was 732 days. TLR and TVR in DCB-iRA and nDES-iRA at 3 years were similar: 15.6% versus 16.3% (*P*=0.99) and 15.6% versus 23.3% (*P*=0.38). In 41 well-matched lesion pairs after propensity score analysis, the cumulative incidence of TLR and TVR in DCB-iRA and nDES-iRA at 3 years was 12.9% versus 16.3% (P=0.70) and 12.9% versus 26.1% (P=0.17), respectively. On QCA analysis, although the acute gain was smaller in DCB-iRA (0.85 mm versus 1.53 mm, P<0.001), the minimum lumen diameter at follow-up was similar (1.69 mm versus 1.87 mm, P=0.29). The late lumen loss was lower (0.09 mm versus 0.52 mm, P=0.009) in DCB-iRA.

**Conclusions:**

DCB-iRA is feasible for CAC.

## 1. Introduction

Despite the improved outcomes of new generation drug-eluting stents (nDES) [1], a previous report shows that there are still more ischemic events and higher bleeding complications with the treatment of patients with severe coronary artery calcification (CAC) [2].

Innovative strategies are required for the treatment of CAC [3]. Drug-coated balloons (DCB) are one of the leading-edge devices that transfer antiproliferative drugs into the lesions via single balloon inflation to prevent restenosis, with nothing implanted in the coronary artery tree [4]. A recent study that compared outcomes of the treatment of de novo coronary artery lesions with DCB and nDES showed similar safety and long-term efficacy [5, 6]. In addition, we recently reported that the clinical outcomes after DCB treatment of moderate or severe calcified lesions and noncalcified lesions were similar [7].

However, CAC is associated with larger dissections after high-pressure balloon dilatation, with more stents consequently required for bailout or due to unsatisfactory results [8]. Thus, aggressive lesion debulking with rotational atherectomy (RA) is occasionally performed to avoid large dissections followed by subsequent high-pressure balloon dilatation [9], although the use of a large burr has not been recommended due to high complications [10].

On the other hand, the efficacy of intravascular imaging devices in PCI was recently reported [11] and these imaging devices are recently used to guide RA (intravascular image-guided RA: iRA) [3, 12].

The aim of this study was to assess the safety of aggressive debulking with iRA and the long-term efficacy of DCB angioplasty following iRA. Furthermore, we compared DCB with nDES after iRA in patients with CAC.

## 2. Methods

### 2.1. Study Population

From April, 2014, to April, 2017, 123 consecutive patients (166 lesions) were evaluated with severely calcified de novo coronary artery lesions who underwent an iRA followed by DCB angioplasty (DCB-iRA group: 54 patients, 68 lesions) or nDES implantation (nDES-iRA group: 69 patients, 98 lesions) at Matsunami General Hospital (Gifu, Japan).

The patients included in this study had CAC that appeared on angiography as radio-opaque regions without cardiac motion before contrast injection and generally involved both sides of the arterial wall. Only lesions were included in this study that met one of the following criteria: (1) the lesion was not crossable by the smallest balloon or by intravascular ultrasound (IVUS) or optical frequency domain imaging (OFDI) (70 lesions); (2) the lesion could not be dilated with a high-pressure balloon or a scoring balloon (27 lesions); or (3) the lesions had extensive intimal deposition of calcium ( the arc of calcium > 270°) assessed by an imaging catheter [13] (69 lesions).

Patients with acute coronary syndrome, restenotic lesions, thrombotic lesions, in-stent restenosis and bypass graft lesions were excluded. All patients provided written informed consent. The study complied with the Declaration of Helsinki for investigation in human beings and was approved by the institutional ethics committee of our institution.

### 2.2. Intravascular Image-Guided RA and the Choice of Adjunctive Therapy after RA

Patients were pretreated with daily doses of 100mg of aspirin and 75mg of clopidogrel or 3.75mg of prasugrel. A 300-mg loading dose of clopidogrel or a 20-mg loading dose of prasugrel was administered before the procedure if patients had not been pretreated at least 4 days earlier. Heparin was administered to maintain an activated clotting time of > 300 seconds during the procedure. All cases were treated with IVUS guidance (View IT, 35MHz and AltaView, 40MHz; Terumo Corp. Tokyo, Japan or Opticross: Boston Scientific, Natick, MA, USA) or OFDI guidance (FastView™ and LUNAWAVE™, Terumo Corp.).

RA (Rotablator system, Boston Scientific) was performed by experienced operators. The baseline burr rotational speed was set at 195,000rpm for a 1.25mm burr and 175,000 rpm for larger burrs (1.5mm - 2.25mm burr) before passing the burr into the guiding catheter. An “oscillating ablation technique” [14] was used that slowly and gently engaged the lesion with the rotating burr, which was then slowly retracted (not pecking quickly) to limit the rotational speed from baseline (<5,000 rpm) during any run (<15 seconds). The operators were highly recommended to advance the burr as slowly and smoothly as possible, by 1-2mm under fluoroscopy, while discerning lesion contours and borders by contrast injection. The operator monitored pitch changes, advancer knob resistance and drive shaft vibration. The operators also controlled the guidewire bias to avoid excessive load on the burr and rpm decreases, setting the optimal ablation vector of the burr by retracting the guidewire to a proximal position or using a burr of smaller size, if needed. Although selection of DCB or nDES after an iRA was left to the discretion of the operator, the decision was made before iRA to investigate a crossover between the strategies.

The only brand of DCB used was the SeQuentPlease (B. Braun, Berlin, Germany). In the nDES-RA group, the stents implanted included 39 Xience (Abbott Laboratories, Abbott Park, IL), 23 Ultimaster / 3 Nobori (Terumo Corp.), 12 Premiere / 4 Synergy (Boston Scientific) and 17 Resolute (Medtronic Inc., Santa Rosa, California).

### 2.3. Burr Selection: Lesion Preparation with iRA before DCB Angioplasty

A stepped-burr approach was used, with the maximum burr size determined by IVUS or OFDI images (not by angiography), while considering guidewire bias. The target maximum burr size was set at 0.7 of the mean reference diameter (MRDI) obtained by intracoronary imaging. After every rotablation, intracoronary imaging was performed to decide whether to use a larger burr.

Then, DCB was inflated at low pressure (1-4 atm) for 30 to 60 seconds to avoid major dissections, while confirming by angiography to ensure that the balloon was in contact with the vessel luminal wall as well as estimated by changes in the ST segment of ECG. In order to ensure sufficient contact of the balloon surface with the vessel luminal wall at low pressure, the balloon diameter was the same or ≥0.25 mm of the MRDI, and the balloon length exceeded the target lesion at both sides by at least 2mm.

### 2.4. Burr Selection: Lesion Preparation with iRA before nDES Implantation

A maximum burr size was also set at 0.7 of the MRDI. Before stenting after an iRA, the lesion was dilated with a high-pressure balloon or a scoring balloon until full expansion of the balloons was achieved at 12-22atm. If necessary, high-pressure balloon dilatation was further undertaken to treat stent underexpansion.

### 2.5. Outcomes

The study end points were target lesion revascularization (TLR) and target vessel revascularization (TVR). Acute procedural success, defined as residual stenosis <50% and stenosis reduction of at least 20% defined by angiography, procedural complications, death and major adverse cardiac events (MACE) were also investigated. All end points were defined according to the Academic Research Consortium (ARC) definitions [15].

### 2.6. Quantitative Coronary Angiography

The latest coronary angiograms, which were obtained at 6 months or later after PCI, were analyzed to obtain follow-up data. The angiograms before and after PCI and at follow-up were analyzed using the QAngio XA Version 7.3 (MEDIS Medical Imaging Systems BV, Leiden, the Netherlands).

### 2.7. Statistical Analysis

Continuous variables are presented as mean and standard deviation (SD) or median and interquartile range (IQR) and categorical variables as counts and percentages. Student's t-test and Chi-squared test were used for comparisons. A* P* value of < 0.05 was considered significant.

The cumulative incidence rates of TLR and TVR in the two groups were derived from Kaplan–Meier analyses, and the log-rank test was used to compare the differences between the groups. Cox proportional hazard models were used to compare the unadjusted outcomes between the groups, and the results are presented as hazard ratios (HR) with 95% confidence intervals (CI). To adjust for differences in baseline characteristics between the two procedures, propensity score matching was performed with a greedy matching algorithm. The matching algorithm used a multivariate logistic regression model that included baseline covariates with P < 0.05 in univariate analysis as well as chronic total occlusion (CTO) and the minimum lumen diameter (MLD) before procedure as established predictors [16]. All statistical analysis was performed by R software version 3.4.1(2017-06-30).

## 3. Results

### 3.1. Characteristics of Patients and Lesions

The baseline characteristics of the patients and lesions are summarized in Table 1. The right coronary artery was the target vessel more often in the nDES-iRA group, whereas the left circumflex coronary artery was more often in the DCB-iRA group. Ostial lesions and bifurcation lesions were more frequent in the DCB-iRA group. The reference diameter was significantly smaller in the DCB-iRA group (2.28 ± 0.58 mm versus 2.49 ± 0.55 mm, respectively, P=0.019) and the lesion length was significantly shorter in the DCB-iRA group (11.89 ± 6.41 mm versus 18.78 ± 7.91 mm, respectively, P<0.001).

### 3.2. Characteristics of the Procedural Devices and Acute Procedural Results

The DCB-iRA group had a significantly higher mean maximum burr size (1.74 ± 0.28 mm versus 1.66 ± 0.22 mm, respectively, P=0.038) and a higher angiographic burr/artery ratio (B/A ratio) (0.79 ± 0.17 versus 0.69 ± 0.13, respectively, P<0.001) (Table 2).

The acute success rates were high and major complications were rare in both groups. The incidence of coronary flow disturbance after rotablation was 14.8% in the DCB-iRA group and 11.6% in the nDES-iRA group (P=0.12). However, most coronary flow disturbances were due to TIMI 2 slow flow despite a high B/A ratio. Crossover and major dissections were rare; one patient (1.9%) in the DCB-iRA group experienced major dissection (NHLBI type E) just after rotablation (B/A ratio, 0.83) and crossover to DES (Table 3).

### 3.3. Clinical Follow-Up

**Figure 1:**
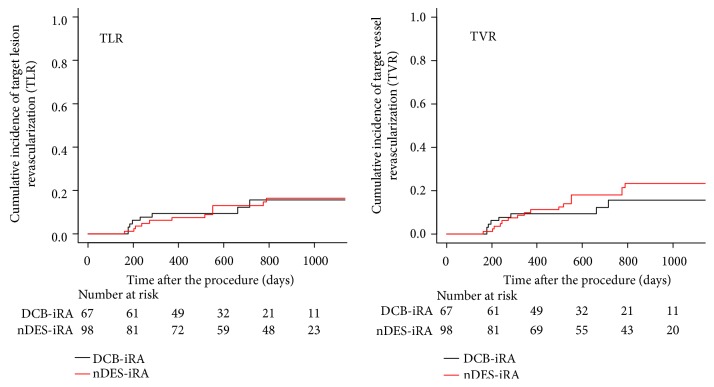
Cumulative Kaplan–Meier estimates of the incidence of target lesion revascularization (TLR) (left panel) and target vessel revascularization (TVR) (right panel), using the crude cohorts of 68 lesions treated with drug-coated balloons (DCB) after intravascular image-guided rotational atherectomy (iRA) (DCB-iRA) and 98 lesions treated with new generation drug-eluting stents (nDES) after iRA (nDES-iRA).

The median (IQR) follow-up was 732 (484-1,030) days. The cumulative incidence rates of TLR at 1, 2, and 3 years were not significantly different between the DCB-iRA and nDES-iRA group (9.3% versus 6.2% and 15.6% versus 12.9%, and 15.6% versus 16.3%, respectively, P=0.99) (HR 1.01, 95%CI 0.41-2.50, P=0.99). In addition, there was no significant difference between the DCB-iRA group and nDES-iRA group in the cumulative incidence rates of TVR at 1, 2 and 3 years (9.3% versus 10.0%, 15.6% versus 18.0% and 15.6% versus 23.3%, respectively,* P*=0.38) (HR 0.69, 95%CI 0.30-1.60, P=0.39) (Figure 1).

**Figure 2:**
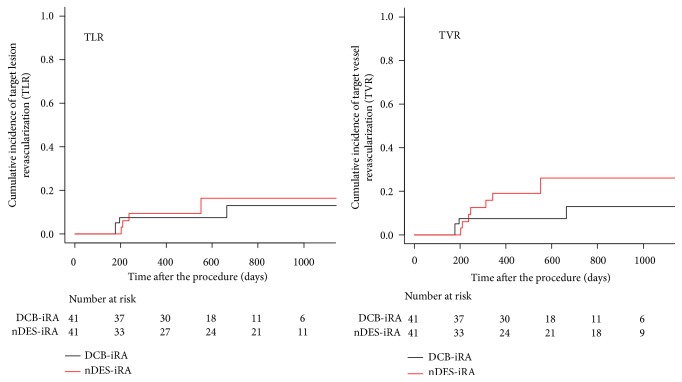
Cumulative Kaplan–Meier estimates of the incidence of target lesion revascularization (TLR) (left panel) and target vessel revascularization (TVR) (right panel), using the propensity matched cohorts of 41 lesions treated with drug-coated balloons (DCB) after intravascular image-guided rotational atherectomy (iRA) (DCB-iRA) and 41 lesions treated with new generation drug-eluting stents (nDES) after iRA (nDES-iRA).

The propensity score was calculated from target vessel, ostial lesion, bifurcation lesion, reference diameter, and lesion length as covariates with P < 0.05 in univariate analysis as well as CTO and MLD before the procedure. In the propensity score-matched group (41 lesions in each group, Table 4), there was still no significant difference between the DCB-iRA group and nDES-iRA group in the cumulative incidence rates of TLR at 1, 2, and 3 years (7.5% versus 9.3% and 12.9% versus 16.3% and 12.9% versus 16.3%, respectively, P=0.70) (HR 0.77, 95%CI 0.21-2.88, P=0.70). The cumulative incidence rates of TVR at 1, 2, and 3 years in the DCB-iRA group (7.5%, 12.9%, and 12.9%, respectively) were also not different from those in the nDES-iRA group (19.2%, 26.1%, and 26.1%, respectively, P=0.17) (HR 0.44, 95% CI 0.13-1.47, P=0.18) (Figure 2).

During the follow-up period, there were 10 cardiac deaths (2/54, 3.7% in the DCB-iRA group and 8/69, 11.6% in the nDES-iRA group) and 9 noncardiac deaths (6/54, 11.1% in the DCB-iRA group and 3/69, 4.3% in the nDES-iRA group).

At 1, 2, and 3 years, there were no significant differences between the DCB-iRA group and the nDES-iRA group in the cumulative rates of all-cause mortality (7.6% versus 13.1%, 10.5% versus 14.9% and 20.9% versus 14.9%, P=0.95), cardiac death (1.9% versus 8.8%, 1.9% versus 10.7% and 9.4% versus 10.7%, respectively, P=0.18), and MACE (15.1% versus 23.3%, 21.7% versus 28.2% and 31.6% versus 30.2%, respectively, P=0.47).

There were no definite cases of vessel or stent thrombosis in either group, although one case of possible stent thrombosis (1/69, 1.4%) and 2 cases of probable stent thrombosis (2/69, 2.9%) were observed only in the nDES-iRA group.

### 3.4. Quantitative Angiographic Analysis

Follow-up angiography was performed for 38 patients (71.7%) with 51 lesions (76.1%) in the DCB-iRA group and 46 patients (66.7%) with 62 lesions (63.3%) in the nDES-iRA group.

The results of quantitative coronary angiography are shown in Table 5. After propensity matching, the DCB-iRA group and the nDES-iRA group had a similar reference diameter (2.46 ± 0.64 mm versus 2.37 ± 0.39 mm, respectively, P=0.53), lesion length (15.01 ± 6.67 mm versus 15.77 ± 7.25 mm, respectively, P=0.70), and B/A ratio (0.74 ± 0.11 versus 0.72 ± 0.11, respectively, P=0.45).

At intervention, the acute gain (AG) in lumen diameter was smaller in the propensity matched DCB-iRA group than in the nDES-iRA group (0.85 ± 0.37 mm versus 1.53 ± 0.43mm, respectively, P<0.001), and the post-PCI percent diameter stenosis was larger in the propensity matched DCB-iRA group than in the nDES-iRA group (24.10 ± 10.0% versus 9.80 ± 6.46%, respectively,* P*<0.001). However, at follow-up, the minimum lumen diameter (1.69 ± 0.57 mm versus 1.87 ± 0.60 mm, respectively, P=0.29) and the percent diameter stenosis (29.52 ± 17.62% versus 23.81 ± 20.52%, respectively, P=0.29) were similar between the two propensity matched groups, and the late lumen loss (LLL) and loss index (LLL/AG) in the DCB-iRA group was significantly lower than that in the nDES-iRA group (0.09 ± 0.48 mm versus 0.52 ± 0.63 mm, respectively, P=0.009, and 0.03 ± 0.52 versus 0.32 ± 0.39, respectively, P=0.026).

## 4. Discussion

The method in the present study is based on the hypothesis that sufficient plaque debulking without barotrauma is a better approach for complex lesions [9, 17].

On this hypothesis, the prospective randomized trial (STRATAS) was performed to investigate the outcome of 500 patients randomized to either an aggressive rotablation strategy (maximum burr/artery ratio >0.7 followed by no angioplasty, or angioplasty ≤1 atm) versus routine rotablation (maximum burr/artery ratio ≤0.7, followed by routine balloon angioplasty ≥4 atm) [9]. MACE and emergent CABG were less in aggressive RA than in routine RA (2.0% versus 4.0%), coronary perforation occurred only in the routine group, and severe flow disturbance occurred in only 1.2% in the aggressive RA. Nonetheless the aggressive rotational atherectomy strategy offered no advantage over more routine burr sizing plus routine angioplasty in MLD at follow-up, loss index (0.62 for the aggressive strategy versus 0.54 for the routine strategy) and restenosis rate. Therefore, since the advent of the first-generation DES, a B/A ratio has been recommended to be set at 0.5 to 0.6 before implantation of DES [3].

However, even in new generation DES era, CAC has been reported to be associated with higher procedural complications and an increased risk of MACE in patients undergoing not only PCI but also CABG [2, 18]. More innovative strategies are required for the treatment of CAC.

To the best of our knowledge, this is the first report to assess the effect of DCB angioplasty at low pressure avoiding barotrauma after an aggressive rotablation of severely calcified lesions.

The acute results of aggressive iRA in the present study showed that this procedure was safe, with less complication and less bailout stenting. Although the targeted maximum bur size was set at 0.7 of MRDI and a high B/A ratio was obtained, procedural complications were rare and only one crossover from DCB to DES was observed (1.9%). A severe flow disturbance (TIMI 0) was observed in only 2 cases (1.6%). Since the incidence of flow disturbance was reported to be 0-3.8% with a contemporary approach [3, 12], it is reasonable to suppose that aggressive iRA (oscillating ablation technique by the burr of 0.7MRDI) is as safe as rotablation when the B/A ratio is set at 0.5 to 0.6 by angiography.

In addition, with respect to long-term efficacy, the loss index in the DCB-iRA group in the present study was smaller than that in the aggressive RA-low pressure POBA group in the STRATAS trial (0.08 versus 0.62, respectively) [9]. Furthermore, it was found that the DCB-iRA group has an equivalent TLR and TVR rate to the nDES-iRA group, as well as after propensity matching.

In the SeQuentPlease World Wide Registry, the TLR rate after DCB only for de novo lesions was similar to that after DCB plus bare metal stents (BMS) (1.0% versus 2.4%, respectively), whereas the TVR rate after DCB only tended to be smaller than that of DCB plus BMS (1.0% versus 3.6%, respectively, P=0.09) [19]. However, this tendency for a difference in the TVR rate was not found in the present study (12.9% in the DCB-iRA versus 26.1% in the nDES-iRA, P=0.17).

Of note, Rissanen et al. recently reported that a stentless strategy with DCB following balloon dilatation or cutting balloon dilatation after RA for CAC was safe and effective (TLR / MACE at 1 and 2 years were 1.5% / 14% and 3.1% / 20%, respectively) [20]. Compared with their results, the TLR rates in our study were higher although MACE was similar (9.3% / 15.1% and 15.6% / 21.7%, respectively). This is attributed to the fact that the target lesions were in larger vessels in their study, and routine noninvasive testing and follow-up angiographies were not performed. In contrast, the reference diameter in our study was smaller (2.28±0.58 mm). Moreover, 61.1% were diabetic mellitus patients and 27.9% of the lesions were ostial lesions in our study. Since the predictors of restenosis are a history of diabetes mellitus, a small reference diameter, and ostial lesions [21], the outcome obtained is considered to be clinically acceptable. In addition, it should be noted that bailout stent was needed in 10% of the procedures in their study, while alternatively only one bailout stent (1.9% of the patients) was needed in the present study.

Although there was no difference between the two groups in the reference diameter or B/A ratio after propensity matching, the results of acute angiography showed that the DCB-iRA group had significantly less acute gain, lower post-PCI MLD, and higher residual stenosis. The differences in acute gain and residual stenosis between the groups are attributed to the expansion of the lumen during stenting. In contrast, at follow-up angiography, there was no difference between the groups in residual stenosis or MLD. The reason for this was a significantly larger late lumen loss (LLL) in the nDES-iRA group than in the DCB-iRA group (0.52 mm versus 0.09 mm, respectively,* P*=0.009). The LLL in the stented group was larger than what has been reported previously for the stenting of noncalcified lesions [22]. We speculate that this is due to residual calcium causing damage to the stent polymer or chronic stent recoil, even after preparation of the lesions with rotablation. Additionally, further barotrauma was incurred by the high-pressure balloons.

On the other hand, given that the SeQuentPlease balloon is folded, a majority of the drug is protected by the folds during delivery to the calcified lesions. The reported washout of a delivered and retracted noninflated balloon is 6% [23]. With appropriate drug delivery during balloon inflation, drug uptake into the lesion is sufficient to inhibit LLL and reduce the TLR rate.

Several potential advantages of stentless strategy using DCB over stenting have been suggested [5, 19]: (1) a short duration of dual antiplatelet therapy (DAPT) of 1 month is feasible, and DAPT can be discontinued at any time if there are bleeding events; (2) the absence of a metal scaffold to deliver antiproliferative drug maintains vasomotion and may prevent neoatherosclerosis in the treated artery; (3) finally and most important, stentless PCI with low pressure DCB dilation is often simpler and less time consuming, especially for complex anatomy such as calcified ostial lesions, bifurcation lesions and diffuse long lesions.

### 4.1. Study Limitations

There are several limitations in this study. First, the two procedures were assigned in a nonrandomized manner. We conducted propensity score matching to minimize the difference in patient characteristics. However, there may still be residual selection bias and confounding, and the number of propensity matched QCA pairs was small. Second, this study was a retrospective, small study performed at a single center. Since a relatively small number of the patients were enrolled and only a small number of events occurred due to small sample size, the present study may have been underpowered to determine the relationship between the incidence of TLR/TVR and DCB treatment after iRA. A study in a larger population is needed to definitively define the effects of DCB angioplasty.

### 4.2. Conclusion

For severely calcified coronary lesions, a stentless strategy utilizing DCB angioplasty at low pressure following iRA is feasible and effective.

## Figures and Tables

**Table 1:** Baseline characteristics of patients and lesions.

Variables	DCB-iRA lesions (n=68)	nDES-iRA lesions (n=98)	*P* value
Number of patients	54	69	
Age (years)	71±9	71±9	0.75
Male (%)	38 (70.4)	45 (65.2)	0.57
Smoking (%)	10 (18.5)	20 (29.0)	0.21
Diabetes (%)	33 (61.1)	39 (56.5)	0.71
Hypertension (%)	38 (70.4)	53 (76.8)	0.54
Hyperlipidemia (%)	38 (70.4)	43 (62.3)	0.44
Chronic kidney disease (%)	19 (35.2)	28 (40.6)	0.58
Hemodialysis patients (%)	12 (22.2)	19 (27.5)	0.54
Previous MI (%)	17 (31.5)	20 (29.0)	0.84
Previous CABG (%)	7 (13.0)	6 (8.7)	0.56
PAD (%)	15 (27.8)	21 (30.4)	0.84
Target vessel, n (%)			
LMT	2 (2.9)	5 (5.1)	0.001
LAD	38 (55.9)	47 (48.0)	
RCA	9 (13.2)	35 (35.7)	
LCX	19 (27.9)	11 (11.2)	
Lesion anatomy			
Type B2/C (%)	67 (98.6)	95 (96.9)	0.62
Ostial (%)	19 (27.9)	14 (14.3)	0.047
Bifurcation (%)	46 (67.6)	46 (47.9)	0.016
CTO (%)	3 (4.4)	11 (11.2)	0.16
Quantitative angiography			
Reference diameter (mm)	2.28±0.58	2.49±0.55	0.019
Lesion length (mm)	11.89±6.41	18.78±7.91	<0.001
MLD			
Pre-intervention (mm)	0.87±0.35	0.90±0.41	0.65
Percent diameter stenosis			
Pre-intervention (%)	60.9±12.5	64.5±16.3	0.12

CABG, coronary artery bypass graft surgery; CTO, chronic total occlusion; DCB, drug-coated balloon; LAD, left anterior descending artery; LMT, left main trunk; LCX, left circumflex artery; MI, myocardial infarction; MLD, minimun lumen diameter; nDES, new generation drug-eluting stents; PAD, peripheral artery diseases; RCA, right coronary artery; SD, standard deviation.

**Table 2:** Device characteristics.

	DCB-iRA lesions (n=68)	nDES-iRA lesions (n=98)	*P* value
Number of patients	n=54	n=69	
Sheath size, French(F)			
6F (%)	32 (59.3)	53 (76.8)	0.11
7F (%)	10 (18.5)	9 (13.0)	
8F (%)	12 (22.2)	7 (10.1)	
Approach			
via radial artery (%)	36 (66.7)	44 (63.8)	0.67
via brachial artery (%)	0 (0.0)	2 (2.9)	
via femoral artery (%)	18 (33.3)	23 (33.3)	
Maximum burr size (%)			
1.25mm	5 (7.4)	8 (8.2)	0.18
1.5mm	19 (27.9)	34 (34.7)	
1.75mm	27 (39.7)	45 (45.9)	
2.0mm	7 (10.3)	7 (7.1)	
2.15mm	1 (1.5)	1 (1.0)	
2.25mm	9 (13.2)	3 (3.1)	
Mean burr size, mm	1.74±0.28	1.66±0.22	0.038
B/A ratio	0.79±0.17	0.69±0.13	<0.001

B/A ratio, burr-to-artery ratio.

**Table 3:** Acute procedural outcomes.

	DCB-iRA patients (n=54)	nDES-iRA patients (n=69)	*P* value
Procedure success (%)	53 (98.1)	68 (98.6)	1.0
Patient success (%)	54 (100)	68 (98.6)	
Crossover (%)	1 (1.9)	0	1.0
Major complications			1.0
Death (%)	0	1 (1.4)	
MI (%)	0	1 (1.4)	
CABG (%)	0	0	
Congestive heart failure (%)	0	2 (2.9)	
Minor complications			1.0
Ventricular fibrillation (%)	0	1 (1.4)	
Side branch occlusions (%)	0	1 (1.4)	
RA complications			
Perforation (%)	0	0	1.0
TIMI 0 slow flow (%)	0	2 (2.9)	0.12
TIMI 1 slow flow (%)	0	2 (2.9)	
TIMI 2 slow flow (%)	8 (14.8)	4 (5.8)	
Dissection; NHLBI classification			
A (%)	1 (1.9)	0	0.08
C (%)	1 (1.9)	0	
E (%)	1 (1.9)	0	

CABG, coronary artery bypass graft surgery; MI, myocardial infarction; RA rotational atherectomy.

**Table 4:** Baseline characteristics of patients and lesions in the propensity-matched cohorts.

Variables	DCB-iRA lesions (n=41)	nDES-iRA lesions (n=41)	*P* value
Number of patients	34	35	
Age (years)	70±8	72±9	0.63
Male (%)	24 (70.6)	20(57.1)	0.32
Smoking (%)	6 (17.6)	10 (28.6)	0.39
Diabetes (%)	22 (64.7)	17 (48.6)	0.23
Hypertension (%)	24(70.6)	28 (80.0)	0.41
Hyperlipidemia (%)	22 (64.7)	22(62.9)	1.00
Chronic kidney disease (%)	13 (38.2)	14 (40.0)	1.00
Hemodialysis patients (%)	10 (29.4)	12 (34.3)	0.80
Previous MI (%)	11 (32.4)	7 (20.0)	0.28
Previous CABG (%)	3 (8.8)	1(2.9)	0.36
PAD (%)	10 (29.4)	12 (34.3)	0.80
Target vessel			
LMT (%)	2 (4.9)	2 (4.9)	0.79
LAD (%)	23 (56.1)	21 (51.2)	
RCA (%)	9(22.0)	13(31.7	
LCX (%)	7 (17.1)	5 (12.2)	
Lesion anatomy			
Type B2/C (%)	41(100)	39 (95.1)	0.60
Ostial (%)	10 (24.4)	10(24.4)	1.0
Bifurcation (%)	24 (58.5)	23 (56.1)	1.0
CTO (%)	3 (7.3)	5 (12.2)	0.71
Quantitative angiography			
Reference diameter (mm)	2.44±0.63	2.39±0.42	0.66
Lesion length (mm)	14.29±6.84	14.67±6.00	0.79
MLD			
Pre-intervention (mm)	0.93±0.39	0.87±0.35	0.43
Percent diameter stenosis			
Pre-intervention (%)	61.43±14.47	64.67±15.34	0.33
Mean burr size (mm)	1.78±0.32	1.66±0.18	0.028
B/A ratio	0.76±0.14	0.71±0.12	0.10

Abbreviations are the same for Table 1.

**Table 5:** Pre- and postprocedural quantitative angiographic characteristics in the crude and matched cohorts.

	DCB-iRA lesions	nDES-iRA lesions	*P* value
*Crude cohorts*	n=51	n=62	
*At PCI*			
Reference diameter (mm)	2.33±0.63	2.50±0.57	0.14
Lesion length (mm)	11.56±6.19	18.57±7.98	<0.001
MLD (mm)			
Pre-intervention (mm)	0.86±0.38	0.91±0.40	0.49
Post-intervention (mm)	1.75±0.50	2.57±0.51	<0.001
Percent diameter stenosis			
Pre-intervention (%)	62.27±13.31	64.70±15.39	0.38
Post-intervention (%)	22.86±10.11	9.59±6.10	<0.001
Acute Gain (mm)	0.90±0.39	1.66±0.53	<0.001
Rotablator burr size			
Mean burr size (mm)	1.74±0.28	1.68±0.21	0.21
B/A ratio	0.78±0.17	0.70±0.14	0.006
*At follow-up*			
MLD (mm)	1.68±0.63	2.03±0.84	0.015
% diameter stenosis (%)	29.01±20.25	25.12±23.56	0.36
Late lumen loss (mm)	0.08±0.43	0.54±0.80	<0.001
Loss index	0.05±0.48	0.33±0.45	0.002
*Matched cohorts*	n=26	n=26	
*At PCI*			
Reference diameter (mm)	2.46±0.64	2.37±0.39	0.53
Lesion length (mm)	15.01±6.67	15.77±7.25	0.70
MLD (mm)			
Pre-intervention	0.94±0.36	0.86±0.34	0.42
Post-intervention	1.78±0.42	2.38±0.34	<0.001
Acute Gain (mm)	0.85±0.37	1.53±0.43	<0.001
Percent diameter stenosis			
Pre-intervention (%)	60.78±13.27	65.48±15.44	0.24
Post-intervention (%)	24.10±10.00	9.80±6.46	<0.001
Rotablator burr size			
Mean burr size (mm)	1.78±0.32	1.67±0.20	0.16
B/A ratio	0.74±0.11	0.72±0.11	0.45
*At follow-up*			
MLD (mm)	1.69±0.57	1.87±0.60	0.29
% diameter stenosis (%)	29.52±17.62	23.81±20.52	0.29
Late lumen loss (mm)	0.09±0.48	0.52±0.63	0.009
Loss index	0.03±0.52	0.32±0.39	0.026

B/A ratio, burr-to-artery ratio; MLD, minimum lumen diameter; SD, standard deviation; PCI, percutaneous coronary intervention. Loss index; Late lumen loss / Acute gain.

## Data Availability

The data used to support the findings of this study are available from the corresponding author upon request.
